# Comparing the Sensitivity and Specificity of Computed Tomography and Ultrasound in the Diagnosis of Acute Cholecystitis in a Rural Setting

**DOI:** 10.7759/cureus.80316

**Published:** 2025-03-09

**Authors:** Lucas Gerbasi, Tanja Gunsberger, Anthony Santarelli, John Ashurst

**Affiliations:** 1 Arizona College of Osteopathic Medicine, Midwestern University, Glendale, USA; 2 Surgery and Anesthesia, Arizona College of Osteopathic Medicine, Midwestern University, Glendale, USA; 3 Emergency Medicine, Kingman Regional Medical Center, Kingman, USA

**Keywords:** cholecystitis, computed tomography, sensitivity, specificity, ultrasound

## Abstract

Background

Acute cholecystitis (AC) is a common inflammatory disease of the gallbladder, primarily caused by gallstones or sludge blockage. Early diagnosis and treatment are crucial for reducing morbidity and mortality. Ultrasound (US) and computed tomography (CT) are commonly used imaging methods, with US being considered the gold standard. However, recent studies have shown that CT has higher sensitivity and specificity for diagnosing AC in large hospital settings.

Objective

This study aims to determine the sensitivity and specificity of US and CT for AC in a community hospital and to assess the sensitivity and specificity of specific signs seen on US and CT for AC.

Methods

A retrospective cohort study was conducted, including patients who underwent US of the right upper quadrant (RUQ) and/or CT of the abdomen and pelvis, followed by pathological evaluation of the gallbladder after surgical removal between May 1, 2019, and April 30, 2023. Data collected included patient demographics, laboratory values, symptoms, US findings, CT findings, and pathology results. Imaging signs were recorded based on radiology reports and were considered positive if any sign was present. A true positive for CT and US was recorded if imaging was positive for AC and the pathological report confirmed AC.

Results

A total of 187 patients who underwent cholecystectomy for AC, with a median age of 60.6 years, were included in the final analysis. Abdominal pain was the most common presenting symptom (176/187, 94.1%), followed by nausea (114/187, 61.0%) and vomiting (75/187, 40.1%). White blood cell (WBC) counts were elevated in all groups, with median levels of 11.3 (US only), 15.8 (CT only), and 12.3 (both US and CT) (p<0.001). Most patients (169/187, 90.4%) received an RUQ US, and 123/187 (65.8%) underwent a CT scan prior to surgery. The sensitivity of US and CT was found to be similar (98.6% and 93.4%, respectively) when following a one-sign criterion. US was more sensitive than CT (80.9% and 70.0%, respectively) when a two-sign criterion was used. In a direct comparison between CT and US, US was more sensitive in detecting cholelithiasis and a thickened gallbladder wall (95.9% and 92.3%, respectively), while CT was more sensitive in detecting pericholecystic fluid and gallbladder distension (83.6% and 95.7%, respectively).

Conclusion

In a community emergency department, US had higher sensitivity than CT for detecting AC when a two-sign criterion was used. Based on these results, US should continue to be the first-line imaging modality in patients suspected of having AC.

## Introduction

Acute cholecystitis (AC) is an inflammatory disease of the gallbladder affecting over 200,000 people in the United States annually, with over 90% of cases caused by blockage of the cystic duct by gallstones or sludge [1,2]. The progression of the disease is characterized by edema and congestion of the gallbladder in the first two to four days after blockage, leading to distension. This is followed by a hemorrhagic and necrotic phase between days 3 and 5, caused by compression of the gallbladder due to increased intramural pressure. The purulent phase then follows, characterized by leukocyte infiltration of necrotic tissue [3].

Early diagnosis and treatment of AC are vital for improving patient outcomes and reducing hospital costs. Treatment via cholecystectomy within the first three days of diagnosis has been associated with lower mortality compared to those who underwent surgery on day 5 [4]. In addition, patients diagnosed with AC and treated via cholecystectomy within 24 hours have shown a reduced rate of postoperative complications compared to those initially treated with antibiotics followed by cholecystectomy [5].

A diagnosis of AC is suspected with the presence of one local and one systemic sign, while the addition of an imaging sign confirms a definitive diagnosis. Local signs of AC include a positive Murphy’s sign and right upper quadrant pain/tenderness with or without eating. Systemic signs include fever, elevated C-reactive protein, and/or an elevated white blood cell count. Ultrasound (US) and computed tomography (CT) are the most common imaging modalities used for diagnosing AC in the acute setting. Common US findings supporting the diagnosis include gallstones or sludge, pericholecystic fluid, gallbladder distension, and an edematous or thickened gallbladder wall. CT findings suggestive of AC include gallbladder distension, wall thickening, pericholecystic fat stranding, and/or pericholecystic fluid [6]. Previous meta-analyses on the diagnosis of AC have shown US of the RUQ to have a sensitivity ranging from 81% to 88% and a specificity of 83% to 88% [7,8]. Meanwhile, the sensitivity and specificity of CT for diagnosing AC were found to be 94% and 59%, respectively [8].

While US has traditionally been considered the gold standard for diagnosing AC, many healthcare providers opt for additional imaging to increase diagnostic confidence. Currently, CT is recommended by the American College of Radiology (ACR) for patients presenting to the emergency department with acute abdominal pain [9]. However, the drawbacks of CT compared to US, namely access, cost, and radiation exposure [10], raise questions about its necessity in diagnosing AC. Access to high-quality imaging in community hospitals compared to larger institutions is an important factor when evaluating how new findings impact patient care. Critical access hospitals have been shown to have fewer and lower-quality CT scanners [11]. While CT has demonstrated higher sensitivity than US for diagnosing AC [8], its effectiveness in the community setting remains uncertain. Therefore, the authors of this paper aim to compare US and CT in their effectiveness for diagnosing AC in rural settings.

## Materials and methods

Setting

Kingman Regional Medical Center (KRMC) is a 235-bed hospital located in northern Arizona, serving approximately 55,000 emergency department patients per year. The surgery department has four board-certified general surgeons and is actively involved in undergraduate and graduate medical education. KRMC houses emergency medicine and family medicine residency programs. Family medicine residents rotate with general surgeons during their second year of training, and surgical residents from an external general surgery residency began rotating through the department monthly in 2020.

Protocol

Following institutional review board (IRB) approval, a retrospective cohort of patients diagnosed with AC between May 1, 2019, and April 30, 2023, was reviewed for inclusion. To be included in the final analysis, patients must have undergone both US of the RUQ and computed tomography of the abdomen and pelvis (CTAP) with a pathological evaluation of the gallbladder following surgical removal, or had a US or CTAP of the RUQ with a pathological diagnosis following surgical removal. Patients who did not meet this criterion or who were transferred from an outside facility were excluded from the final analysis.

All US and CT scans were interpreted by a board-certified radiologist, and the final pathology of the gallbladder was verified by a board-certified pathologist, with results documented in the electronic medical records. Additionally, all US imaging was performed in an imaging suite at KRMC by certified sonography technicians. All data were abstracted by trained research staff, who underwent training on proper data abstraction prior to data collection. Training was conducted by the primary investigator, who worked directly with the research assistant to ensure compliance with the protocol and accuracy of data collection. With adherence to a quality-controlled protocol and a structured abstraction tool, research assistants manually collected all data points [12]. Data abstracted included baseline patient demographics, presenting laboratory values, symptoms, US findings, CTAP findings, and pathology results.

Imaging signs were recorded as either present or absent based on the radiology report in the medical record. If the report was not definitive, such as stating that a particular sign was "probable," it was considered positive. CT signs that were recorded included a distended gallbladder, thickened gallbladder wall (>3 mm), gallstones, and pericholecystic fluid/edema and/or fat stranding [13]. US signs that were recorded included the presence of a sonographic Murphy’s sign, a distended gallbladder, a thickened gallbladder wall (>3 mm), gallstones, and pericholecystic fluid/edema. A US or CT scan was recorded as positive if any sign was present and negative if all signs were absent [13]. A true positive for CT and US was recorded if the imaging modality had at least one sign for AC and the final pathological report confirmed AC.

Data analysis was performed using IBM SPSS Statistics for Windows, Version 27 (Released 2020; IBM Corp., Armonk, New York), with statistical significance defined as P ≤ 0.05. Results are reported descriptively with point estimates and a measure of distribution. The Kruskal-Wallis test, followed by a Mann-Whitney U test, was used to assess continuous data.

## Results

A total of 187 patients were included in the final analysis, with a median age of 60.6 years (42.8-72.2 years). Ninety-four of 187 (50.3%) were male, and 159 of 187 (84.5%) self-identified as White (Table 1). Of the 187 cholecystectomies performed, 169 of 187 (90.4%) received an RUQ US, and 123 of 187 (65.6%) received a CT scan prior to surgery. In total, 64 of 187 (34.2%) patients underwent US only, 18 of 187 (9.6%) underwent CT only, and 105 of 187 (56.1%) received both US and CT as part of their initial workup for abdominal pain. The median age of patients who underwent US only was 48.5 years (35.5-69.1 years), those who underwent CT only had a median age of 71.2 years (62.4-80.0 years), and those who underwent both US and CT had a median age of 63.0 years (49.7-73.2 years) (p < .001).

**Table 1:** Demographics of Patients US: ultrasound; CT: computed tomography; IQR: interquartile range.

Demographics	Total (n = 187)	US (n = 64)	CT Only (n = 18)	Both US and CT (n = 105)	P
Age: median (IQR 25%-75%)	60.6 (35.5-69.1)	48.5 (62.4-80.0)	71.2 (49.7-73.2)	63 (49.7-73.2)	< .001
						Sex (m)	50.3% (94/187)	34.4% (22/64)	44.4% (8/18)	61.0% (64/105)	
White	84.5% (159/187)	81.3% (52/64)	100.0% (18/18)	83.8% (88/105)	

Abdominal pain was the most common presenting symptom, occurring in 176 of 187 patients (94.1%), and was present across all subgroups (Table 2). Nausea was the second most prevalent symptom, reported in 114 of 187 patients (61.0%), and was more common in those who underwent CT only compared to those who underwent US only (13 of 18 (72.2%) vs. 35 of 64 (54.7%), respectively). Vomiting was the third most common presenting symptom, occurring in 76 of 187 patients (40.1%). Diarrhea (17 of 187 (9.0%)) and fever (10 of 187 (5.3%)) were the least common presenting symptoms in those diagnosed with AC.

**Table 2:** Presenting Symptoms of Patients US: ultrasound; CT: computed tomography.

Presenting Symptoms	Total (n = 187)	US (n = 64)	CT Only (n = 18)	Both US and CT (n = 105)
Abdominal pain	94.1% (176/187)	93.8% (60/64)	94.4% (17/18)	94.3% (99/105)
Nausea	61.0% (114/187)	54.7% (35/64)	72.2% (13/18)	62.9% (66/105)
Vomiting	40.1% (75/187)	40.6% (26/64)	50.0% (9/18)	38.1% (40/105)
Fever	5.3% (10/187)	6.3% (4/64)	16.7% (3/18)	2.9% (3/105)
Diarrhea	9.0% (17/187)	7.8% (5/64)	16.7% (3/18)	8.6% (9/105)

White blood cell (WBC) count levels were found to be elevated (12.3; 9.45-15.2), exceeding the normal range (4.5-11 × 10⁹/L) (Table 3). The median WBC levels for US only, CT only, and both US and CT were 11.3 (8.6-14.0), 15.8 (11.05-20.55), and 12.3 (9.2-15.4), respectively, with a statistically significant difference (p < .001). Lipase levels had a median value of 69.0 (36.5-101.5). There were notable distinctions among the three groups (US only, CT only, and both US and CT), with median levels of 69.5 (38.0-101.0), 50.0 (29.5-70.5), and 73.0 (30.5-115.5), respectively (p = .020). AST (33.0; 22.0-44.0), ALT (27.0; 11.5-42.5), total bilirubin (0.8; 0.5-1.1), alkaline phosphatase (91.0; 69.5-112.5), lipase (69.0; 36.5-101.5), and lactate (1.5; 1.15-1.85) median levels did not exhibit any significant differences between the subgroups.

**Table 3:** Lab Values of Patients US: ultrasound; CT: computed tomography; IQR: interquartile range.

Lab Values	Total (n = 187): Median (IQR 25%-75%)	US (n = 64): Median (IQR 25%-75%)	CT Only (n = 18): Median (IQR 25%-75%)	Both US and CT (n = 105): Median (IQR 25%-75%)	P
White blood cell count, n=185	12.3 (9.45-15.2)	11.3 (8.6-14.0)	15.8 (11.05-20.55)	12.3 (9.2-15.4)	< .001
						Aspartate aminotransferase, n=185	33.0 (22.0-44.0)	32.0 (23.0-41.0)	35.0 (23.5-46.5)	33.0 (20.5-45.5)	0.687
												Alanine aminotransferase, n=185	27.0 (11.5-42.5)	26.5 (9.5-43.5)	25.0 (1.5-48.5)	28.0 (14.5-41.5)	0.682
Total bilirubin, n=184	0.8 (0.5-1.1)	0.8 (0.5-1.1)	0.95 (0.7-1.2)	0.8 (0.45-1.15)	0.36												
						Alkaline phosphatase, n=185	91.0 (69.5-112.5)	86.0 (66.0-106.0)	106 (89.0-123.0)	95.0 (70.5-119.5)	0.233						
												Lipase, n=166	69.0 (36.5-101.5)	69.5 (38.0-101.0)	50.0 (29.5-70.5)	73.0 (30.5-115.5)	0.02
Lactate, n=73	1.5 (1.15-1.85)	1.35 (1.1-1.6)	1.8 (1.35-2.25)	1.6 (1.25-1.95)	0.071												

Cholelithiasis (137 of 169; 81.1%) and a thickened gallbladder wall (131 of 169; 77.5%) were the most common US findings in patients with AC (Table 4). Among those positive for AC upon pathological evaluation, cholelithiasis was found in 115 of 137 (83.9%), and a thickened gallbladder wall was found in 109 of 131 (83.2%). Pericholecystic fluid collection (63 of 169; 37.9%) and an enlarged/distended gallbladder (32 of 169; 19.0%) were the least common US findings. However, among specimens positive for AC, pericholecystic fluid collections were observed on US in 59 of 63 cases (93.7%), and an enlarged/distended gallbladder was found in 29 of 32 cases (90.6%).

**Table 4:** Ultrasound Findings in Patients With Acute Cholecystitis

Ultrasound Signs	n (%), N = 169	% Present on Positive Pathology
Sonographic Murphy sign	64 (37.9%)	85.9% (55/64)
Thickened gallbladder wall	131 (77.5%)	83.2% (109/131)
Enlarged/distended gallbladder	32 (19.0%)	90.6% (29/32)
Cholelithiasis	137 (81.1%)	83.9% (115/137)
Pericholecystic fluid collection	63 (37.9%)	93.7% (59/63)

Among patients who underwent CT prior to cholecystectomy, pericholecystic fluid/fat stranding was observed in 73 of 123 (59.3%), and cholelithiasis was seen in 68 of 123 (55.3%) (Table 5). However, an enlarged/distended gallbladder (53 of 58; 91.4%) and a thickened gallbladder wall (55 of 63; 87.3%) were the most common pathological findings following cholecystectomy in those who underwent CT as part of the diagnostic workup.

**Table 5:** Computed Tomography Findings in Patients With Acute Cholecystitis

Computed Tomography Signs	n (%), N = 123	% Present on Positive Pathology
Cholelithiasis	68 (55.3%)	85.3% (58/68)
Thickened gallbladder wall	63 (51.2%)	87.3% (55/63)
Pericholecystic fluid/fat stranding	73 (59.3%)	89.0% (65/73)
Enlarged/distended gallbladder	58 (47.2%)	91.4% (53/58)

The sensitivity of US was found to be higher than CT when the number of AC signs on imaging was considered (Table 6). When using the criterion of one sign being present, the sensitivity was 98.58% for US and 96.36% for CT. When requiring two signs to be present, the sensitivity was 80.85% for US and 70.0% for CT. The specificity of CT (7.69%) was found to be higher than US (3.57%) when only one AC imaging sign was present. However, the specificity of CT was lower than US when two or more signs of AC were present on imaging.

**Table 6:** Sensitivity/Specificity of Ultrasound and Computed Tomography Based on Number of Signs Present US: ultrasound; CT: computed tomography.

	1 Sign		2 Signs		3 Signs		4 Signs		5 Signs
	US	CT	US	CT	US	CT	US	CT	US
Sensitivity	98.58%	96.36%	80.85%	70.00%	53.90%	36.36%	23.40%	6.36%	2.13%
Specificity	3.57%	7.69%	17.86%	15.39%	71.43%	53.85%	89.29%	84.62%	100%

For the subgroup that received both CT and US (Table 7), cholelithiasis was detected on both modalities 60.7% of the time (51 of 84 cases). Meanwhile, a thickened gallbladder wall, pericholecystic fluid, and an enlarged gallbladder were detected on both modalities in 50.6% (50 of 90), 45.7% (32 of 70), and 20.0% (8 of 40) of cases, respectively.

**Table 7:** Prevalence of Imaging Signs for Acute Cholecystitis for Computed Tomography and Ultrasound (N=105) US: ultrasound; CT: computed tomography.

	Present on US Only	Present on CT only	Present on Both
Pericholecystic fluid	15.7% (11/70)	38.6% (27/70)	45.7% (32/70)
Cholelithiasis	32.1% (27/84)	7.1% (6/84)	60.7% (51/84)
Enlarged/distended gall bladder	5.0 % (2/40)	75.0% (30/40)	20.0% (8/40)
Thickened gall bladder wall	37.8% (34/90)	6.7% (6/90)	50.6% (50/90)

In a comparison of signs detectable on both CT and US, sensitivity and specificity were calculated for each sign in patients who received both CT and US (Table 8). CT demonstrated higher sensitivity than US in detecting pericholecystic fluid (83.60% vs. 65.57%) and gallbladder distension (95.65% vs. 41.30%). Meanwhile, US showed higher sensitivity in detecting cholelithiasis (95.90% vs. 64.38%) and a thickened gallbladder wall (92.30% vs. 61.54%). Specificity was found to be higher in US for pericholecystic fluid (66.67%) and cholelithiasis (27.27%).

**Table 8:** Sensitivity and Specificity of Overlapping Signs (N=105) US: ultrasound; CT: computed tomography; TGW: thickened gallbladder wall; PCF: pericholecystic fluid; GBD: gallbladder distension.

	PCF		Cholelithiasis		GBD		TGW	
	US	CT	US	CT	US	CT	US	​​​​​​​CT
Sensitivity	65.57%	83.60%	95.90%	64.38%	41.30%	95.65%	92.3%	61.54%
Specificity	66.67%	11.11%	27.27%	9.09%	60.0%	0	0	25.0%

The sensitivity and specificity of two specific signs were compared in patients who received both US and CT (Table 9). For US, the combination of a thickened gallbladder wall plus gallstones yielded the highest sensitivity (57.6%). Meanwhile, for CT, the combination of a thickened gallbladder wall plus pericholecystic fluid had the highest sensitivity (33.7%). Specificity was highest for US in the combinations of gallbladder distension plus gallstones (92.3%) and gallbladder distension plus pericholecystic fluid (100%). For CT, specificity was highest with the combination of a thickened gallbladder wall plus distension (84.6%).

**Table 9:** Sensitivity and Specificity of Two Sign Combinations (n=105) US: ultrasound; CT: computed tomography; TGW: thickened gallbladder wall; GBD: gallbladder distension; PCF: pericholecystic fluid.

	TGW + GBD		TGW + PCF		TGW + Cholelithiasis		GBD + PCF		GBD + Cholelithiasis		PCF + Cholelithiasis	
	US	CT	US	CT	US	CT	US	CT	US	CT	US	CT
Sensitivity	14.13%	18.95%	41.30%	33.70%	57.61%	25.0%	10.87%	30.44%	13.04%	23.91%	32.61%	27.17%
Specificity	84.62%	84.62%	76.92%	53.85%	38.46%	46.15%	100%	69.23%	92.3%	69.23%	84.62%	53.85%

## Discussion

This retrospective study is among the first to evaluate the sensitivity and specificity of CT and US for the diagnosis of AC at a community hospital. The patients in the current study were slightly younger than those who underwent cholecystectomy in previous reports [14,15]. Given that the study location lies within a rural county in Arizona, this difference in age could be due to health disparities that exist within rural counties, leading to a younger population presenting with the same disease [16]. Those who received CT were significantly older than those who received only US. These results align with the common trend of CT being beneficial as the first imaging modality for elderly patients admitted for surgical assessment [17]. The frequencies of presenting signs were consistent with previous studies of gallstone disease, with RUQ tenderness and vomiting being the most common [18]. The prevalence rates of fever, defined as a temperature above 99.5°F [19], and diarrhea show consistency with previous studies, indicating their poor sensitivity and specificity in the diagnosis of AC [20]. No single clinical lab value or sign can be solely relied upon for the diagnosis of AC [21]. However, leukocytosis (>11,000 WBC) was present in every subgroup and was significantly higher in those who received CT only and both CT and US. A high WBC count in the subgroups that received CT is expected, as the ACR recommends CT in the presence of an elevated WBC count and a negative US [9].

When assessing a single imaging sign for the diagnosis of AC [13], there was no meaningful difference in sensitivity and specificity between US and CT, with both having very high sensitivity and very low specificity. The sensitivity of US was found to be higher than reported by Fagenholz et al. (98% vs. 79%). These higher rates could be due to the cohort in this study, which included only those who had a cholecystectomy for confirmation by pathology and did not include those who may have undergone percutaneous gallbladder drainage or were treated via pharmaceutical management. When assessing two imaging signs for the diagnosis of AC, US was found to have a higher sensitivity than CT (81% vs. 70%), which does not support the existing literature that shows CT having a higher sensitivity [15]. The evaluation of the combination of specific two signs shows high specificity in the diagnosis of AC. While prior literature utilizes any two signs as a criterion for diagnosing AC, employing a more defined criterion specifying which two signs must be present could prove advantageous in ruling out AC.

CT and US were directly compared in their diagnostic abilities using a cohort that had both CT and US performed (n = 105). In the evaluation of the sensitivity and specificity of individual signs, typical trends were followed for the sensitivity of cholelithiasis between US and CT, with this study showing 95.9% and 64.38%, respectively, compared to 96% and 75%, respectively [22]. This difference in sensitivity is due to gallstones being composed of cholesterol (20%), which, when surrounded by bile, shows minimal hypoattenuation on CT [23]. US was found to have a higher sensitivity than CT in detecting TGW, which could be due to the difficulty of differentiating subserosal edema from surrounding ascites on CT [24]. However, CT had better sensitivity in detecting distension, but US had better sensitivity in detecting pericholecystic fluid/edema than CT. This split in sensitivity for different signs suggests the value in using CT and US in complement with each other when the results of one are uncertain.

The usage of CT as a diagnostic tool for abdominal pain has significantly increased over the past two decades [25]. In the current study, CT was performed first in only 43.8% (82/187) of all patients, despite emerging literature showing higher sensitivity for CT in the diagnosis of AC. This may be a result of the training programs at the hospital, where residents receive continuous education regarding guidelines, imaging appropriateness, quality improvement, and supervision from faculty [26]. Current guidelines suggest CT as a potential first-line imaging modality for abdominal pain with an unknown etiology and advocate for its use when additional imaging is necessary if US is negative (ACR). However, the current study shows that CT offers no improvement in sensitivity when a one-sign criterion is used and is inferior when a two-sign criterion is applied. This information, in conjunction with the current results showing US is performed first, agrees with ACR’s recommendation that US should be the first imaging modality for RUQ pain. US offers benefits such as lower cost to the patient and no exposure to ionizing radiation compared to CT. However, this study also demonstrates differences in the imaging modalities’ ability to detect signs, pointing to the usefulness of using these modalities in combination.

There are several limitations within this study. The cohort included only those treated via cholecystectomy and did not include patients with AC managed with antibiotics or a percutaneous tube. Patients with AC who were not treated with a cholecystectomy may have presented differently in the data collected. Additionally, data were collected only on those with AC and did not include any patients diagnosed with chronic cholecystitis. The diagnostic imaging results were retrieved from existing medical records and were not re-read by a radiologist, leading to the possibility of inconsistency among different radiologists reading the scans.

## Conclusions

The necessity of US in the diagnosis of AC has recently been brought into question, but this study refutes emerging literature suggesting that CT is more sensitive than US in diagnosing AC. Current guidelines recommending US as the first-line imaging modality for RUQ pain and CT as a potential additional imaging tool should continue to be followed. Additionally, when considering signs observed on both CT and US for clinical decision-making, greater reliance should be placed on the imaging modality that demonstrates higher sensitivity for that particular sign.
